# Severe Frostbite on Both Hands and Feet in a Vignette Case: From Physics to Clinics

**DOI:** 10.7759/cureus.29085

**Published:** 2022-09-12

**Authors:** Dzemail Detanac, Sead Marovac, Ilker Sengul, Dzenana Detanac, Demet Sengul, Esma Cinar, Safet Muratovic

**Affiliations:** 1 General Surgery, General Hospital Novi Pazar, Novi Pazar, SRB; 2 Endocrine Surgery/General Surgery, Giresun University Faculty of Medicine, Giresun, TUR; 3 Ophthalmology, General Hospital Novi Pazar, Novi Pazar, SRB; 4 Pathology, Giresun University Faculty of Medicine, Giresun, TUR; 5 Gastroenterology, General Hospital Novi Pazar, Novi Pazar, SRB

**Keywords:** surgical pathology, histopathology, pathology, amputation, surgery, cold injury, frostbite

## Abstract

Frostbite is a cold injury that predominantly affects homeless or intoxicated people, adventurers, and soldiers. It can lead to both superficial tissue damage and tissue necrosis to amputation; thereby leading to significant patient morbidity and disability. The most affected parts of the body for cold injuries are toes, fingers, and exposed facial parts. Of note, these injuries are relatively frequent in the colder climate part of the world, but they can also occur in regions with a warmer climate, during the winter months. We present a vignette case of a 40-year-old male admitted to the department of surgery with severe frostbite lesions on both hands and feet. The aforementioned injury occurred a few months ago, during the winter. During the first hospitalization, immediately after the injury, the patient was initially admitted and treated with conservative therapy with a multidisciplinary approach, to which he did not respond well. To this end, amputation was indicated, which the patient refused. At that time, the patient stopped the treatment and left the hospital. Five months after the injury, he agreed to the amputation treatment. Mummification of fingers of both hands and whole feet was present at the time of the last hospitalization. Amputation was performed with full patient recovery. Better knowledge of frostbite might help in better treatment of the cases.

## Introduction

Frostbite, per se, is a freezing injury, a result of cold exposure in which initial cooling leads to a decrease in blood flow, vasoconstriction, and localized ischemia, histopathologically. Lower temperatures and extended exposure time increase the risk as well as the extent of the injury [1]. The frequency of frostbite increases in wartime and is characteristic of soldiers. In peacetime conditions, they are most often found in mountaineers, but also homeless people, alcoholics, psychiatric patients, and outdoor extreme sports participants [2].

These injuries may be reversible or irreversible, depending on the extent of the exposure and cellular damage, and are classified into four degrees [1-3]. Frostbite can lead to severe damage to the affected areas, dysfunction, and amputation. Long-term sequelae after frostbite injury are also recorded and include neuropathic and nociceptive pain, vasomotor disorders, and frostbite arthritis [4]. Herein, we present an extremely rare case with a fourth-degree frostbite injury of the feet and fingers of both hands, requiring amputation.

## Case presentation

A 40-year-old previously healthy man was admitted to the Department of General Surgery, General Hospital Novi Pazar, Serbia due to frostbite of the feet and fingers of both hands, which occurred a few months before, during the winter of the same year. He was injured at a temperature below 0ºC, when, after voluntarily leaving the reception center for migrants, he stayed outside as a pedestrian for a long time, without data on the length of time during which he was exposed to the direct influence of the cold.

**Figure 1:**
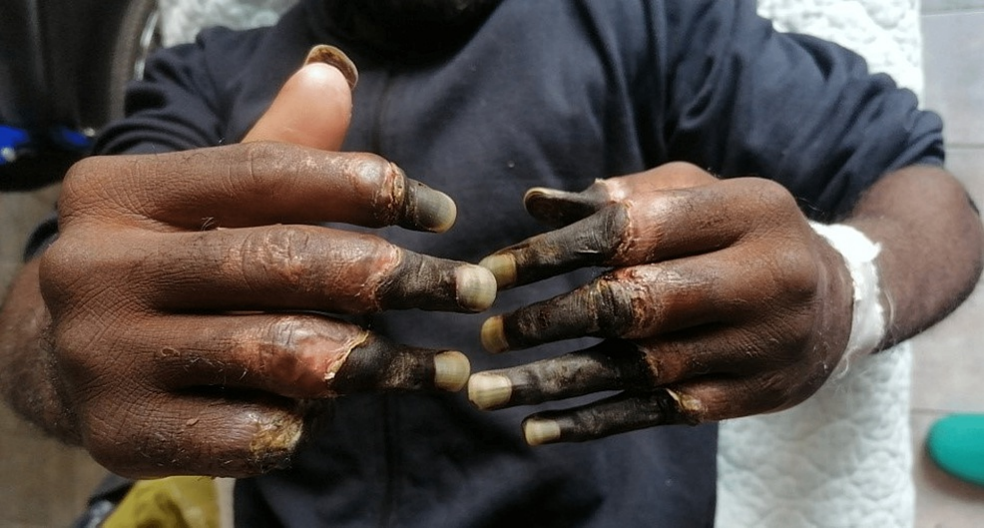
Fourth-degree finger frostbite on both hands.

**Figure 2:**
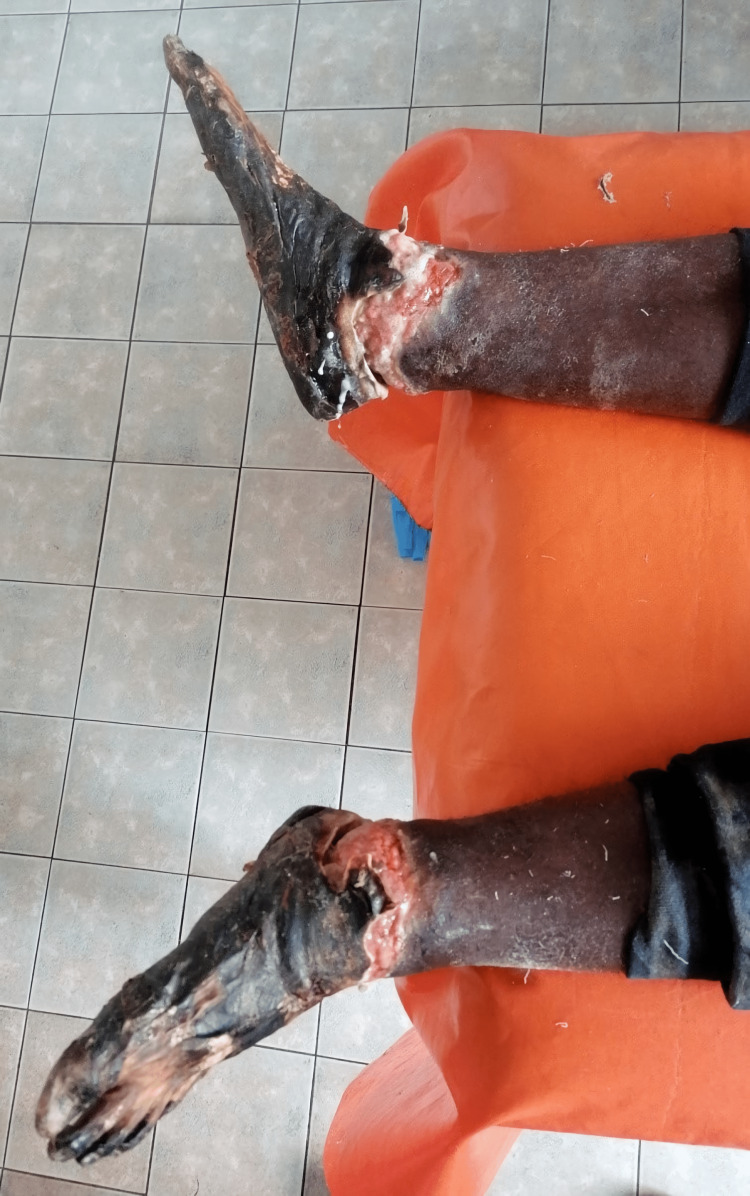
Tissue mummification of both feet due to fourth-degree frostbite.

Since the injury, he has been hospitalized twice, and both times, he stopped the treatment on his own accord and left the hospital. During the first hospitalization, immediately after the injury, signs of frostbite on the hands and feet were recognized. Conservative measures and local treatment of frostbite, according to the protocol, were started immediately, to which the patient did not respond well. The patient continued his treatment in a tertiary health institution, where he was treated by a multidisciplinary specialist team when amputation was proposed. He refused the indicated amputation and left the hospital. Afterward, he came back again because of severe pain in his hands and feet and upset. He had normal vital signs, no capillary refill on both hands, fingers, and feet, with necrosis and tissue mummification of distal and middle phalanges from the second to fifth fingers of the right hand and the distal phalanx of the thumb and distal and middle phalanges from the second to fifth fingers of the left hand (Figure 1). Tissue mummification of the feet due to fourth-degree frostbite was also recognized (Figure 2). The rest of his physical exam was unremarkable. After the Doppler sonography of the hands and feet, the amputation process was planned. To this end, a bilateral below knee amputation was performed in addition to the amputation of the medial and distal phalanx of the second to fifth fingers and the distal phalanx of the thumb of the left hand, and amputation of the distal phalanx of the second and fifth fingers, and the medial and distal phalanges of the third and fourth finger of the right hand. His postoperative period was uneventful and he demonstrated improved symptoms. The patient left the ward on his initiative and stopped his hospitalization condition after two weeks. Rehabilitation therapy had been continued on an outpatient basis for our vignette case.

## Discussion

Frostbite injuries are infrequent and mostly not life-threatening, unless complications such as simultaneous hypothermia, trauma, infections, and sepsis are present, which occur due to loss of the skin barrier and the development of secondary infection and dehydration [5]. However, frostbite injuries can vary from minimal tissue loss to extensive necrosis, leading to amputation with severe morbidities and varying degrees of disability in the patient. The most affected parts of the body are toes, fingers, and facial areas when exposed unprotected to freezing temperatures [5]. The severity of the frostbite is associated with a temperature gradient on the skin surface and the duration of exposure. These injuries are caused by (i) cellular death caused by tissue freezing and (ii) reperfusion injury that occurs during rewarming that triggers a series of histopathological processes that include vasoconstriction, the appearance of intracellular ice crystals, endothelial damage, stasis, thrombosis, and tissue ischemia. Histopathologic alterations might be reversible or irreversible, which depends on the degree of frostbite [6,7]. Comorbidities such as diabetes mellitus, peripheral vascular disease, Raynaud's disease, as well as tobacco and alcohol use, may worsen cyto-histopathological tissue and organ injury [8] in frostbite [1].

Cold thermal energy-induced injuries are relatively common in parts of the world with a colder climate (in northern subarctic regions, which account for 0.8-1.2% of all injuries) and in military personnel [9]. In recent times, the popularity of recreational sports (alpinism, hiking, mountain, ice climbing, etc.), along with staying in cold regions, has become a source of significant storage for cases of frostbite. Approximately 100 cases of severe frostbite had been reported in the United States from 2007 to 2014 [10]. However, to the best of our knowledge, a small amount of data, mostly case reports, have been described in the English-language literature. The diagnosis of frostbite is made by clinical examination. Additional laboratory testing may help detect complications. In-hospital treatment includes warm water baths and intravenous fluids, analgesics, antibiotics, thrombolytic, and vasodilatory therapy in specific cases, wound dressing, and other conservative therapy. Of note, frequent re-examination will reveal signs of compartment syndrome, which indicates urgent surgery [1]. There are insufficient data about the utility of hyperbaric oxygen therapy in frostbite therapy. In cases of complete histopathologic tissue necrosis and mummification of the affected part of the body, amputation is the only treatment of choice in terms of surgical pathology.

## Conclusions

Frostbite is a rare but important cause of morbidity and mortality, which has been incompletely defined by the current literature. We postulate that familiarizing with this severe pathologic condition, treatment modalities, and predictors of treatment outcome would be essential for health providers and physicians, in establishing better therapeutic modalities for frostbite. Here and everywhere in the world, educating the general population on this issue is also necessary to avoid situations like the aforementioned vignette case. This issue merits further investigation.
